# Interleukin-7 Optimizes FOXP3+CD4+ Regulatory T Cells Reactivity to Interleukin-2 by Modulating CD25 Expression

**DOI:** 10.1371/journal.pone.0113314

**Published:** 2014-12-08

**Authors:** Federico Simonetta, Nicolas Gestermann, Stéphane Bloquet, Christine Bourgeois

**Affiliations:** 1 INSERM, U1012, Le Kremlin-Bicêtre, France; 2 Univ Paris-SUD, UMR-S1012, Le Kremlin-Bicêtre, France; 3 Division of Hematology, Department of Medical Specialties, Geneva University Hospitals, Geneva, Switzerland; 4 Animalerie centrale, Faculté de Médecine Paris-Sud, Univ Paris-Sud, Le Kremlin-Bicêtre, France; Institut Pasteur, France

## Abstract

The vast majority of Foxp3 regulatory T cells (Treg) exhibits constitutive expression of CD25 (IL-2Rα), which allows the constitution of the high affinity IL-2Rαβγ receptor, ensuring efficient IL-2 binding by Treg. Maintenance of CD25 expression at Treg surface depends on both cell intrinsic factors and environmental stimuli such as IL-2 itself. Whether other factors can participate to maintenance of CD25 expression *in vivo* is at present unknown. In the present work we demonstrated that IL-7, a gamma-chain cytokine exerting a crucial role in T cell development and homeostasis, is able and necessary to sustain the expression of high levels of CD25 at Treg surface. We demonstrated that, during *in vitro* cultures performed in the absence of IL-2, IL-7 is able to sustain CD25 expression at Treg surface through a transcriptional mechanism. By studying mice in which IL-7 signaling is either genetically impaired or increased and by employing adoptive transfer murine models, we demonstrated that IL-7 is necessary for sustained expression of CD25 at Treg surface *in vivo*. To ascertain the biological impact of IL-7 mediated modulation of CD25 expression, we demonstrated that IL-7 modulation of CD25 expression at Treg surface affected their ability to efficiently bind IL-2 and transduce IL-2 signaling. Finally, we demonstrated that IL-7 dependent modulation of CD25 associated with potentiated IL-2 induced expansion of Treg *in vivo*. Collectively, our results identify IL-7 as a necessary factor contributing to sustained CD25 expression at Treg surface *in vivo* thereby affecting their ability to efficiently react to IL-2.

## Introduction

CD4+FOXP3+ T cells represent a subset of CD4+ T cells crucial for immune-responses regulation and prevention of immune-pathology [1]. CD4 Treg were initially characterized by their constitutive expression of CD25 [1]–[2], the alpha chain of interleukin-2 (IL-2) receptor. Together with the beta chain CD122 and the common gamma chain CD132, CD25 expression allows the constitution of the high affinity IL-2 receptor (IL-2Rαβγ). In accordance to their constitutive CD25 expression, Treg cells are sensitive to IL-2 signaling on which they are dependent for their development [3]–[6], their peripheral homeostasis [7]–[11], and their function [12]–[13]. Importantly, Treg cells do not produce IL-2 [14] and are dependent on consumption of IL-2 produced by effector conventional CD4 T cells [11], [15]. Given the very limited amounts of IL-2 available *in vivo* at the steady state and during the first phases of immune responses, expression of the high affinity IL-2Rαβγ is essential for rapid cytokine binding and signal transduction by Treg, the absence of CD25 resulting in their impaired competitive fitness [3].

Mechanisms regulating CD25 expression at Treg surface are only incompletely understood. FOXP3, a transcription factor essential for Treg development and function [16]–[18] sustains CD25 expression at Treg surface by binding the Cd25 promoter both directly [19]–[21] and through its interaction with other transcription factors such as NFAT [22] and AML1/Runx1 [23]. In addition to cell intrinsic mechanisms, environmental factors modulate CD25 transcription and expression in Treg. IL-2 is the best established soluble factor inducing CD25 transcription on conventional T cells [24]. Regarding Foxp3+ Treg, IL-2 induces this positive feedback regulation on its own receptor through a Stat5 dependent mechanism: inhibition of STAT5 renders Treg unable to up-regulate CD25 upon IL-2 stimulation and displaying impaired function *in vivo* [25]. Reduction in IL-2 availability as a result of genetically disrupted IL-2 production leads to down-regulation of CD25 at Treg surface expression [26]. Similarly, increased proportions of CD25 low Treg in ageing has been associated with low IL-2 availability in aged animals [27]. *In vitro* data suggest that other cytokines of the common gamma-chain family, such as IL-4 and IL-7, could modulate CD25 expression at Treg surface [28] but the *in vivo* relevance of these factors is unknown.

IL-7 plays a major role in T cell development and homeostasis. Treg have been longly been considered to be barely sensitive to IL-7 [29]–[30] in accordance to low levels of expression of CD127, the alpha chain of IL-7 receptor, at their surface [31]–[33]. However, both murine and human Treg cells display significant expression of CD127 at levels sufficient to allow IL-7 signal transduction as revealed by STAT5 phosphorylation [34]–[36]. Moreover, we and others recently demonstrated that, similarly to its action on conventional T cells, IL-7 directly sustains Treg survival *in vitro* [28], [34] and plays an essential role in Treg homeostasis *in vivo* both in secondary lymphoid organs [37]–[38] and at site where Treg exhibit high levels of CD127 expression [39]. We hypothesized that, in addition to its direct effect on survival, IL-7 could in part exert its role on Treg homeostasis in conjunction with IL-2. In the present work we showed *in vitro* that IL-7 is able to sustain CD25 expression at Treg surface in the absence of IL-2 by sustaining CD25 transcription. Moreover, we showed that IL-7 is necessary *in vivo* for sustained expression of CD25 at Treg surface. Finally we demonstrated that IL-7 modulation of CD25 expression influenced Treg ability to efficiently bind IL-2 and transduce IL-2 signaling, and drastically affected IL-2 induced Treg expansion *in vivo.*

## Material And Methods

### Ethics Statement

Mice were kept under specific pathogen free conditions in accordance with institutional guidelines in compliance with French and European animal welfare regulations (agreement B-94-043-12, delivered by the French veterinary authorities). All animal studies were approved by the ethics Committee of University Paris.

Sud and protocols conducted were authorized by French veterinary authorities (licence 94–440) in accordance with French and European guidelines.

### Mice

6 to 8 weeks old C57Bl/6 were purchased from Janvier Labs. Mice deficient for Rag gene (Rag-/-) or for Rag and IL-7 genes (Rag-/-IL-7-/-) were used as hosts for adoptive transfer experiments. Rag-/- IL-7-/- mice were kindly provided by Dr. Benedict Seddon. IL-7Rα-/- mice were purchased from the Jackson Laboratory. IL-7-/- and IL-7 transgenic (IL-7 Tg) mice were kindly provided by Dr. Sophie Ezine. Foxp3/GFP mice were purchased from the Jackson Laboratory. All mice were kept under specific pathogen free conditions and all experiments were performed according to institutional guidelines of the European Community.

### Cell purification

Single cell suspensions were prepared from lymph nodes (pooled inguinal, brachial, axillary and mesenteric) and spleens HBSS containing 2% FCS (PAA Laboratories GmbH). For total CD4 T cells transfer, CD4 T cells were isolated using BD magnetic beads (Becton-Dickinson). For CD4+CD25+ Treg adoptive transfer experiments, CD4+ CD25+ cells were isolated by magnetic beads accordingly to manufacturer instructions (Miltenyi). Cell purity for FOXP3 was >90%. For *in vitro* experiments, CD4+ FOXP3/GFP+ cells were FACS sorted from Foxp3/GFP mice (Jackson Laboratory) on a FACSAria cell sorter (BD Biosciences). Cell purity for FOXP3 was >99%.

### FACS analysis

Extracellular staining was preceded by incubation with purified anti-CD16/32 antibodies (FcgRII/III block, 2.4G2) (eBioscience) to block nonspecific staining. Cells were stained with FITC-, PE-, PECy5-, PECy7-APC- APCAlexa750- or APC-H7-labeled or biotinylated appropriate antibodies including: CD4 (GK1.5); TCRβ (H57-597); CD25 (PC61.5), CD122 (5H4) or appropriate isotype Abs. Streptavidin-FITC, PECy5 or PECy7 were used to develop biotinylated Abs. All Abs were purchased from eBioscience except APC-H7-labeled antibodies (BD Bioscience). Intranuclear FOXP3 staining was performed using eBioscience PE- or APC conjugated FOXP3 staining buffer set (FJK-16s). Six-color flow cytometry was performed with a FACSCanto cytometer (BD Biosciences) and data files were analyzed using FlowJo software (Tree star Inc).

### Real time PCR

Total RNA was extracted with a RNeasy Mini kit (Qiagen) and RNA was converted to cDNA using an Enhanced Avian HS reverse transcriptase–PCR (Sigma-Aldrich). Subsequent real-time PCR was performed using the SsoFast Probes Supermix (Bio-Rad). Primers for detection of murine CD25 (Mm00434261_m1) and Hprt1 (Mm00446968_m1) were purchased as Assays-on-Demand from Applied Biosystems. Samples were run in triplicate on a CFX96 Real-Time PCR Detection System (Bio-Rad).

### CFSE labelling

When required, cell division of transferred T cells was assessed by CFSE labelling (Sigma) using standard methods. Cells were resuspended in PBS in a concentration of 10^7^/ml and incubated with CFSE at final concentration of 5 µM for 10 min at 37°C, followed by two washes in ice cold HBSS containing 10% FCS.

### Cytokines

Recombinant murine IL-2 (rmIL-2) and IL-7 (rmIL-7) were purchased from Immunotools.

### IL-2 binding assay

A Fluorokine assay kit (R&D Systems) was used to evaluate the binding of IL-2 by Tregs cells following manufacturer's instructions. Briefly, cells were incubated with biotinylated IL-2 in 25 µl of serum-free PBS for 30 min on ice. Biotinylated IL-2 was revealed with a PECy7 streptavidin. Binding values were determined by the median fluorescence intensities (MFIs) of the IL-2-biotin-streptavidin complex on CD4+FOXP3+ cells. Measurement of binding of an unrelated biotinylated protein was used as a negative control.

### STAT5 phosphorylation experiments

Splenic cells were incubated for 10 min at 37°C with increasing doses of IL-2 (0, 0.1, 1, 10 ng/ml) (Immuno Tools) and immediately fixed in 2% paraformaldehyde. Cells were made permeable by incubation in 90% methanol and then were stained with CD4, TCRβ, FOXP3 antibody and primary rabbit antibody to phosphorylated STAT5 or isotype control (Cell Signaling) revealed with an anti-rabbit Alexa-647 conjugated secondary antibody. Stat5 phosphorylation was then determined by flow cytometry in CD4+TCRβ+FOXP3+ cells.

### Adoptive transfer experiments

Purified CD4+ CD25+ cells (1×10^6^) were injected into Rag-deficient mice (Rag-/-) or Rag-/- mice that were also deficient for IL-7 (Rag-/- IL-7-/-). Mice were then left untreated or treated with IL-2/anti-IL-2 complexes generated as previously described [40]. Briefly, 1 µg of rmIL-2 was co-incubated with 5 µg of anti-IL-2 mAb (clone JES6-1 purchased from R&D) for 30 min at +37°C. Mice were i.p. injected with IL-2/anti-IL-2 complexes (1 µg plus 5 µg) once daily for three days. Six days after transfer, spleens were harvested and analyzed.

### Statistical analyses

Statistical analyses were performed using unpaired Student T test with Graph Pad Software.

## Results

### IL-7 modulates CD25 expression on regulatory T cells *in vitro*

In order to assess the possibility that CD25 expression on Treg cells can be dynamically regulated by IL-7 availability, we FACS sorted CD4+ FOXP3/GFP+ lymph nodes and spleen cells from FOXP3/GFP mice and cultured them overnight with medium alone or in the presence of IL-7 or IL-2 as control.

**Figure 1:**
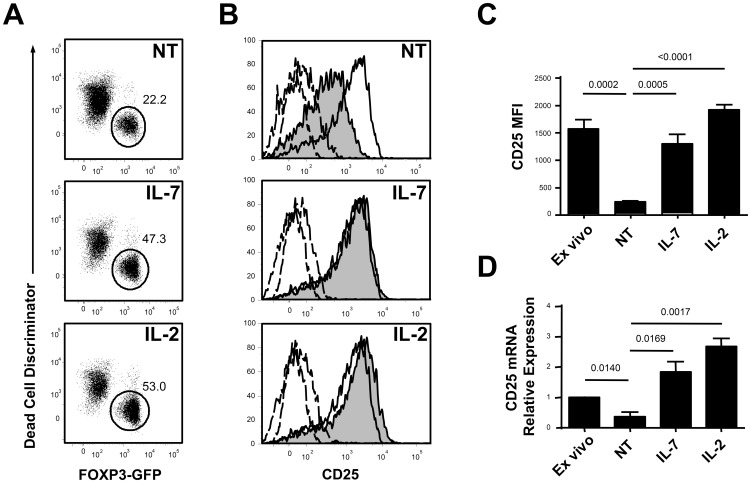
IL-7 signaling dynamically regulated CD25 expression on Treg cells *in vitro*. FACS sorted CD4+ FOXP3/GFP+ and CD4+FOXP3/GFP- cells were incubated overnight untreated (NT) or treated overnight with IL-7 (10 ng/ml) (IL-7) or IL-2 (2 ng/ml) (IL-2). (A) Gating strategy to discriminate live cells. Live cells were defined as Dead Cell Discriminator negative. (B) Representative FACS profiles of CD25 expression on live FOXP3GFP+ after culture are shown. CD25 expression on fresh (open histogram) and incubated (filled histogram) Foxp3/GFP+ cells and isotype control staining are shown. (C) CD25 median fluorescence intensity (MFI) and (D) CD25 mRNA relative expression by PCR. Results are representative of three independent experiments performed in triplicates.

As both IL-2 and IL-7 have been reported by our and other groups to inhibit Treg cell death [28], [34] and in order to study the effect of cytokines on CD25 expression independently from their effect on survival, we restricted our phenotypic analysis to live Treg cells gating as shown in Fig. 1A. As previously described, we found a significant increase in proportions of live Treg cells in cells cultured in the presence of either IL-7 or IL-2 compared to cells cultured in medium alone (data not shown). To further assess whether, in addition to affecting survival, culture in the presence or in the absence of IL-2 and IL-7 could affect cellular metabolic fitness thus resulting in non-specific modulation of protein expression, we analyzed the expression at live Treg surface of CD4, a molecule whose transcription and surface turnover are affected by cellular stress. As shown in S1A Figure, CD4 was expressed at similarly high levels on Treg independently from culture conditions. Looking at CD25 expression, we observed, as expected, a decreased CD25 expression on CD4+ FOXP3+ T cells incubated in the absence of cytokine (Fig. 1B, C) when compared to levels expressed *ex vivo*. As previously reported, the addition of IL-2 to overnight cultures sustained high levels of CD25 expression on Treg cells (Fig. 1B, C). Interestingly, the addition of IL-7 to cultures prevented the decrease in CD25 expression on Treg cells (Fig. 1B, C). We performed similar analysis at the mRNA level to determine whether IL-7 affected CD25 surface expression through a transcriptional mechanism. As expected, CD25 mRNA decreases upon *in vitro* 24 h incubation in the absence of cytokine while addition of IL-2 to culture medium maintained CD25 transcription (Fig. 1D). As demonstrated at the protein level, Treg cells incubated in the presence of IL-7 maintained CD25 transcription (Fig. 1D). To assess whether IL-7 effect on IL-2 receptor subunits was specific to CD25, we next assessed CD122 levels at Treg surface upon culture in the presence of IL-7 or IL-2. We failed to observe any significant difference in CD122 levels between cells cultured in the presence or in the absence of IL-7 (S1A, B Figure). Conversely, in Treg cells cultured with IL-2, receptor occupancy by the cytokine prevented CD122 staining by specific antibodies leading to falsely undetectable levels of CD122 expression (S1A, B Figure). These *in vitro* results demonstrate that, in addition to its positive effects on Treg survival, IL-7 is able to sustain CD25 transcription and surface expression on Treg cells in the absence of IL-2 signaling.

### IL-7 modulates CD25 expression on regulatory T cells *in vivo*

**Figure 2:**
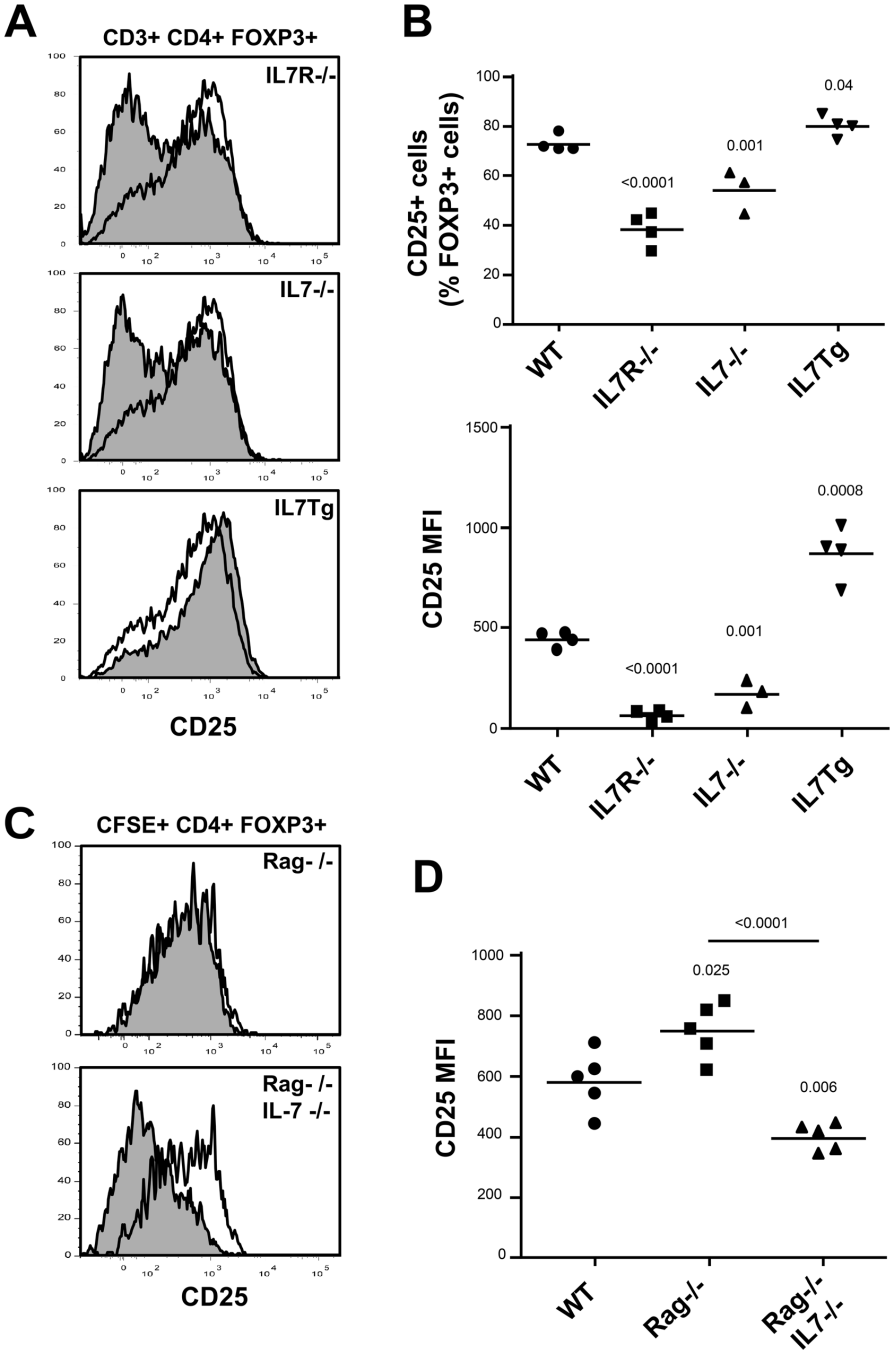
IL-7 signaling dynamically regulated CD25 expression on Treg cells *in vivo*. Analyses of WT, IL-7Rα-/-, IL-7-/- and IL-7Tg mice. Representative FACS profiles (A), percentage and MFI (B) of CD25 expression on splenic CD4+TCRβ+FOXP3+ Treg cells are shown. (A) CD25 expression on Foxp3+CD4 T cells isolated from wild type (open histogram) and mice presenting altered IL-7 pathways (filled histogram). (B) Percentage of CD25 expressing cells and median MFI were determined by substracting isotype control staining to positive expression and to calculate ΔMFI respectively. Data are representative of at least three independent experiments with four to six mice per group. (C, D) Total CFSE stained CD4 T cells were adoptively transferred into either normal B6 mice (WT), IL-7 competent or IL-7 deficient Rag-/- mice. Spleens were harvested after 24 hours and CFSE+CD4+TCRβ+FOXP3+ analyzed for CD25 expression. (C) Representative FACS profiles of CD25 expression on Treg recovered from WT mice (open line) or the indicated Rag deficient mice (gray shaded profile), (D) CD25 expression MFI from one representative experiment are shown. Results are representative of three independent experiments with two to five mice per group. p values of genetically modified mice groups compared to wild-type mice are indicated (unpaired Student's t test).

To confirm the effect of IL-7 on CD25 expression *in vivo*, we first examined CD25 expression on Treg from mice in which IL-7 signaling was disrupted (IL-7Rα-/- or IL-7-/-) or increased (IL-7Tg). We previously reported that IL-7 availability significantly affects Treg cell numbers in these mouse strains in accordance with a role for IL-7 on Treg peripheral homeostasis [34]. After Treg identification as FOXP3 expressing CD4 T cells, we observed lower percentages of CD25 expressing Treg on splenic FOXP3+ CD4+ cells from IL-7Rα-/- or IL-7-/- mice than on wild type cells (WT 72.8%±3.4%, IL-7Rα-/- 38.3%±6.6%, IL-7-/- 54.1%±8.8%), (Fig. 2A and Fig. 2B, upper panel). Conversely, CD25 expression was modestly but significantly increased on splenic Treg cells isolated from IL-7Tg mice when compared to wild type mice (Fig. 2A and Fig. 2B, upper panel). Studies of median of CD25 expression on Treg from the different mouse strains showed equivalent profile: reduced CD25 Median Fluorescence Intensity (MFI) on Treg isolated from IL-7Rα-/- and IL-7-/- mice, whereas a significant increase was detected on Treg from IL-7 Tg mice (Fig. 2B, lower panel). These results indicate that IL-7 quantitatively affects CD25 expression on peripheral CD4+ FOXP3+ T cells *in vivo*.

As IL-7 is essential for thymic development of T cells, including Treg [30], we determined whether IL-7 induced similar effects on wild type Treg *in vivo*. CD4 T cells were isolated from B6 lymph nodes and spleen, CFSE stained and adoptively transferred into either C57Bl/6, RAG-/- or IL-7-deficient RAG-/-IL-7-/- host mice. Injected CD4 T cells were examined in host spleens 18 h after transfer. At this time point, no CFSE dilution was detected among injected CD4 T cells (data not shown), thus confirming the absence of proliferation as previously described [41]–[42]. Treg cells transferred into RAG-/- IL-7-/- but not RAG-/- IL-7 competent hosts significantly down-regulated CD25 surface expression when compared to Treg transferred into either WT (Fig. 2C–D). No CD25 up-regulation was detected on CD4+ FOXP3- cells in all studied hosts (data not shown). Collectively, these *in vivo* experiments support our *in vitro* results and further document that endogenous IL-7 is necessary for maintenance of CD25 expression on Treg cells *in vivo*.

### IL-7 mediated modulation of CD25 expression affects IL-2 binding ability by Treg

**Figure 3:**
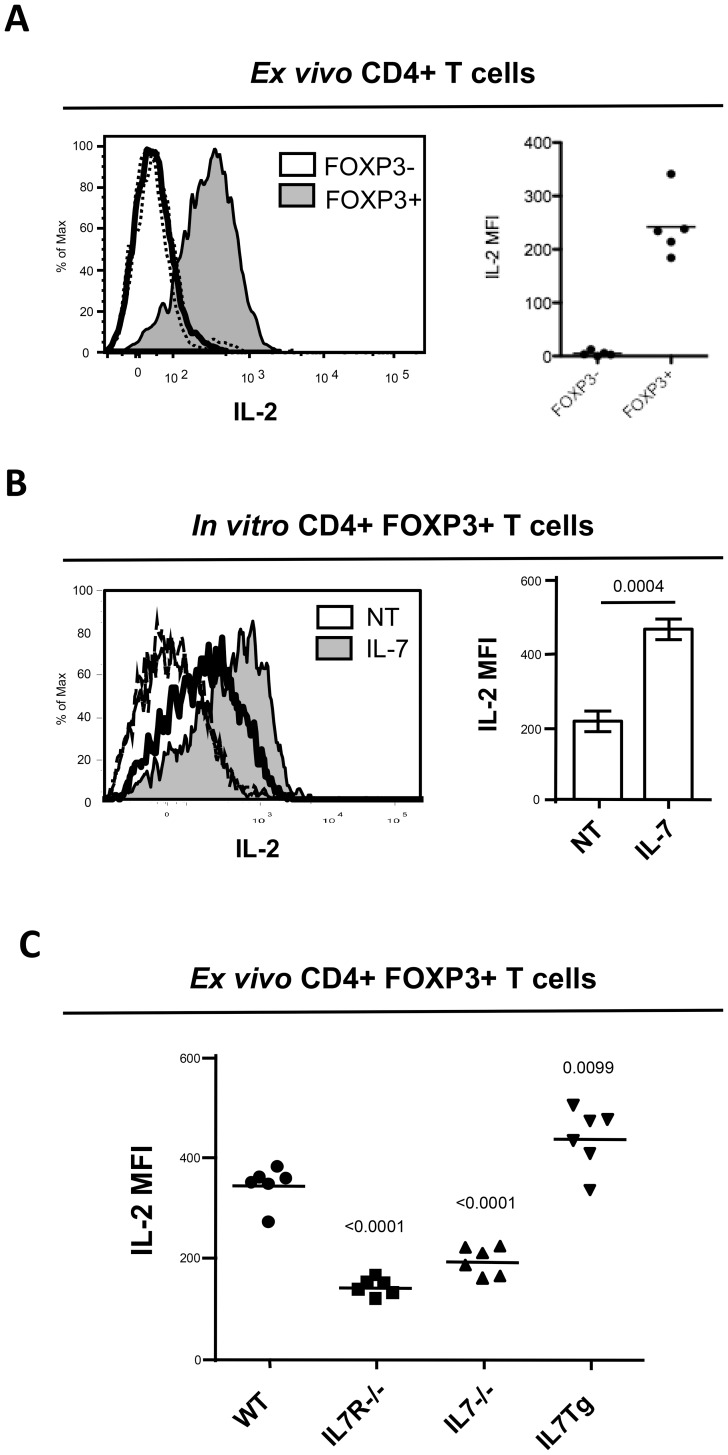
IL-7 availability affected IL-2 binding capacity by Treg. (A) Splenocytes from FOXP3/GFP mice were incubated for 30 min with biotinylated IL-2. Bound biotinylated IL-2 was revealed with fluorochrome-conjugated streptavidine. Shown are representative profiles of IL-2 fluorescence on CD4+GFP- cells (open thick line) or CD4+GFP+ cells (gray shaded profiles). Cells not exposed to biotinylated IL-2 and directly stained with fluorochrome-conjugated streptavidine are shown as negative controls (open thin lines) (B) Total CD4+ cells isolated from FOXP3/GFP mice were cultured in the absence (NT) or in the presence of IL-7 at 10 ng/ml (IL-7). After 24 hours cells were harvested and stained with biotinylated IL-2 as described above. Shown are representative profiles of IL-2 fluorescence on CD4+GFP+ cells cultured with (gray shaded profiles) or without (open thick line) IL-7. Cells not exposed to biotinylated IL-2 and directly stained with fluorochrome-conjugated streptavidine are shown as negative controls (open thin lines). MFI of triplicates ± SD are shown. Data are representative of three independent experiments performed in triplicate. (C) Splenocytes from WT, IL-7Rα-/-, IL-7-/- and IL-7Tg mice were analyzed directly *ex vivo* for IL-2 binding capacity as in (A). Shown are IL-2 MFI on CD4+TCRβ+FOXP3+ cells. Results are representative of two independent experiments with four to six mice per group.

CD25 is necessary for the constitution of IL-2 receptor with the highest affinity and plays a major role in Treg biology. We therefore assessed whether CD25 modulation by IL-7 effectively affected IL-2 binding capacity by Treg. We first studied *ex vivo* IL-2 binding capacity by both conventional FOXP3- and regulatory FOXP3+ CD4 T cells. FOXP3+ Treg efficiently bound IL-2 at their surface (Fig. 3A) accordingly to their constitutive CD25 surface expression. Conversely, FOXP3-conventional CD4 T cells failed to bind detectable levels of IL-2 (Fig. 3A) in agreement to their lack of CD25 surface expression at the steady state. We next assessed IL-2 binding capacity by Treg incubated overnight in the presence or absence of IL-7. As shown in Fig. 3B, prior IL-7 incubation provided enhanced binding capacity for IL-2: a two fold increase in IL-2 fluorokine was detected. To confirm *in vivo* the effect of IL-7 signaling on IL-2 binding capacity by Treg, similar experiments were performed *ex vivo* by analyzing binding of IL-2 fluorokine by splenic Treg isolated from WT, IL-7Rα-/-, IL-7-/- and IL-7 Tg mice. Accordingly to our *in vitro* results, Treg issued from IL-7Rα-/- and IL-7-/- exhibited significantly reduced IL-2 binding capacities compared to WT Treg and conversely Treg from IL-7 Tg mice exhibited significantly higher IL-2 binding capacity (Fig. 3C). Collectively these results demonstrated that IL-7 signaling modulates CD25 expression which affects IL-2 binding capacity by Treg.

### IL-7 signaling optimizes IL-2 signal transduction by Treg

**Figure 4:**
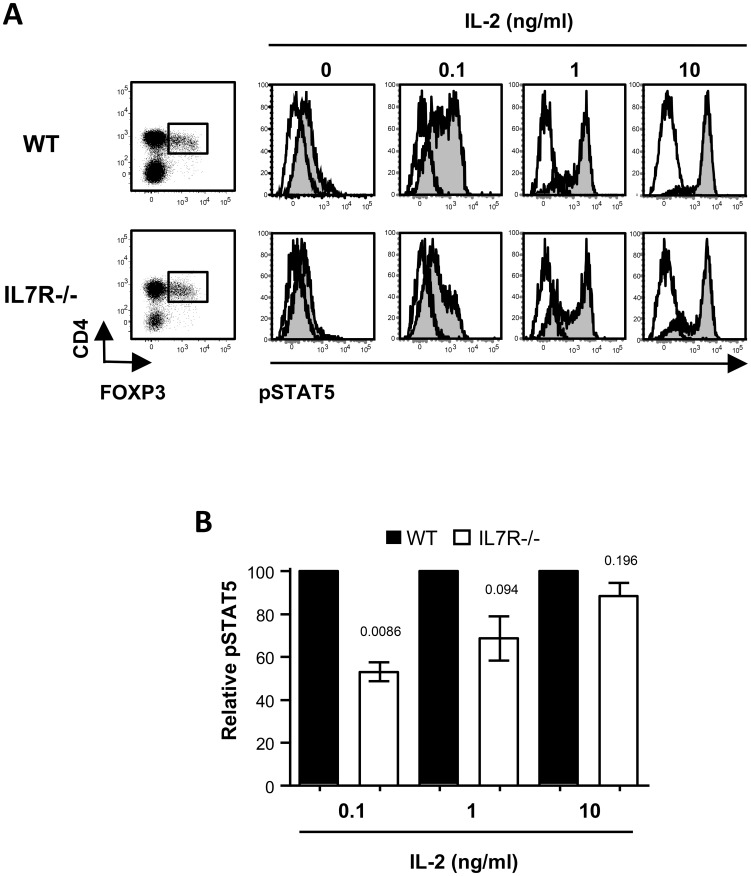
IL-7 signaling affected IL-2 signaling transduction by Treg. WT or IL-7R-/- splenocytes were treated with increasing doses of rmIL-2 for 10 min. Stat5 phosphorylation was then determined by flow cytometry in CD4+TCRβ+FOXP3+ cells. (A) Representative dot plots gated on TCRβ+ lymphocytes showing gating for CD4+FOXP3+ cells and FACS profiles of phospho-STAT5 at different IL-2 doses. Isotype controls are indicated. (B) Relative phospho-STAT5 MFI in IL-7Rα-/- Treg. At any given IL-2 doses, MFI of pSTAT5 staining in IL-7Rα-/- Treg was normalized to pSTAT5 staining in WT Treg. Results are the mean ± SEM from three independent experiments. p values of genetically modified mice groups compared by unpaired Student's t test to wild-type mice are indicated.

We next evaluated whether the decrease in CD25 expression and IL-2 binding capacity observed in Treg in the absence of IL-7 signaling led to a decreased IL-2 signaling transduction. To this aim we assessed STAT5 phosphorylation following short term (10 min) IL-2 incubation on CD4+FOXP3+ splenocytes from WT or IL-7Rα-/- mice. As shown in Fig. 4A, following exposure to IL-2, STAT5 was promptly phosphorylated in WT Treg in a dose dependent manner. Similarly to what observed for IL-2 binding, STAT5 phosphorylation upon IL-2 incubation was exclusively observed in FOXP3+ Treg, while no phosphorylation was detected in FOXP3- conventional CD4 T cells accordingly to their low CD25 expression (S2 Figure). Quantitative analysis of pSTAT5 revealed that IL-7Rα-/- Treg cells presented significantly lower pSTAT5 than WT Treg cells in context of low IL-2 availability (Fig. 4B), indicating that these Treg cells were less responsive to IL-2. Importantly, WT or IL-7Rα-/- mice displayed similar levels of CD122, the β-chain of the IL-2 receptor which plays a major role in IL-2 signal transduction (data not shown). These finding indicates that IL-7 signaling optimizes Treg responsiveness to IL-2 by interfering with CD25 but not CD122 expression.

### IL-7 affects IL-2 induced Treg expansion *in vivo*

**Figure 5:**
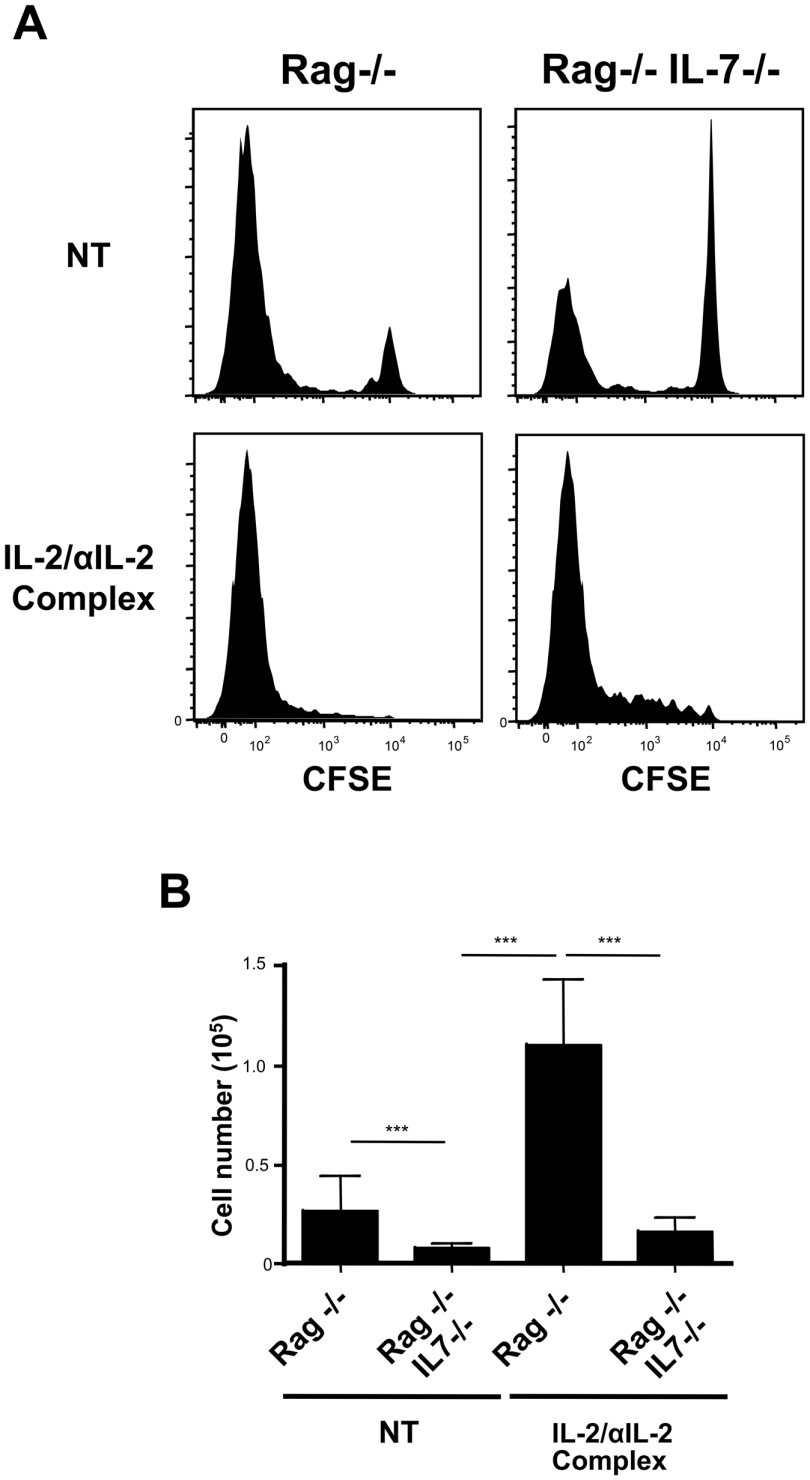
IL-7 signaling is required for IL-2 induced expansion of CD4+CD25+ Treg cells in lymphopenic hosts. CFSE-labeled, purified CD4+CD25+ T cells were transferred into Rag-/- and Rag-/-IL-7-/- mice. Mice were either left untreated or received IL-2/αIL-2 (1 µg/5 µg) complexes at day 1, 2 and 3 after transfer. Spleens from transferred hosts were analyzed at day 6. (A) Representative profiles of CFSE dilution of CD4+TCRβ+FOXP3+ cells. (B) Absolute numbers of splenic CD4+TCRβ+FOXP3+ cells recovered per mouse at day 6. Results are represented as the mean ± SD of 4–5 mice pooled from three independent experiments.

Although not strictly required for Treg proliferation during lymphopenia [9], IL-2 administration in this setting has been shown to strongly increase Treg expansion [10]. Zhang et al. have suggested that IL-2 and lymphopenia could somehow synergize to induce Treg expansion. As lymphopenia is typically characterized by increased levels of IL-7 [43] and having shown that IL-7 availability increases Treg reactivity to IL-2, we speculated that IL-7 could affect IL-2 induced Treg expansion. We transferred purified Treg cells into Rag deficient mice either competent or deficient for IL-7 that were additionally treated or not with IL-2/αIL-2 complexes at d1, 2 and 3. Treg cell recovery at day 1, established before the first IL-2 injection, allowed confirming that similar initial number of Treg were present in both IL-7 deficient and competent hosts prior to IL-2 complex injection (data not shown). At day 6, we determined CD4+ FOXP3+ Treg proliferation profiles and cell numbers. As we previously reported [37], CD4+ FOXP3+ Treg proliferation was detected both in IL-7 deficient host compared to IL-7 competent hosts (Fig. 5A) but Treg expansion was significantly increased in IL-7 competent hosts, accordingly to a role for IL-7 for Treg expansion in empty hosts in the absence of IL-2. Similarly, IL-2/αIL-2 complexes injection induced strong Treg proliferation in both IL-7 competent or IL-7 deficient Rag-/- mice (Fig. 5A) suggesting IL-7 availability did not affect significantly IL-2 dependent Treg proliferation. We next analyzed Treg recovery at day 6: IL-2 mediated effect on Treg expansion was essentially detected in IL-7 competent hosts whereas low Treg numbers were present in the absence of IL-2/αIL-2 complexes injection but also in IL-7 deficient hosts treated with IL-2/αIL-2 complexes. IL-2 induced Treg expansion was approximately four-fold reduced in IL-7 deficient compared to IL-7 competent mice (Fig. 5B). Collectively, these results indicate that, although not being necessary for Treg proliferation in response to IL-2, IL-7 modulates IL-2 induced Treg expansion in lymphopenic hosts.

## Discussion

IL-2 represents the principal factor regulating Treg function and homeostasis. Both at steady state and during immune responses, IL-2-producing conventional CD4 T cells represent the main source of IL-2 employed by Treg [11]. Numbers of Treg and IL-2 producing CD4 T cells are finely associated in a quorum sensing-like system [15]. However, IL-7 has been recently reported by our and other groups as a complementary factor affecting Treg homeostasis [37]–[39]. In the present report we unraveled an interplay between these two cytokines, demonstrating *in vivo* that IL-7 availability affects CD25 expression and IL-2 usage by murine Treg.

CD25 levels at Treg surface are dictated by both cell intrinsic factors, such as FOXP3 expression, and environmental stimuli, mainly IL-2 signaling. IL-2 exerts its positive feedback regulation on its own receptor through STAT5 mediated CD25 transcription in both conventional T cells [24] and Treg cells [25]. In addition, IL-2 signaling sustains, specifically in Treg, FOXP3 expression which in turn induces CD25 transcription [19]–[20]. We showed here that IL-7 is both sufficient and necessary to maintain CD25 expression at Treg surface *in vivo.* Such IL-7 dependent modulation of CD25 expression develops through a transcriptional mechanism. Molecular pathways involved in this process were not investigated in this report. However similarly to IL-2, IL-7 has been reported to induce STAT5 phosphorylation [34]–[36] and to influence FOXP3 expression [28] in Treg, thus acting on both major pathways involved in maintenance of CD25 expression at Treg surface. Importantly such potentiating role of IL-7 on CD25 expression has been previously described on activated conventional T cells [44]–[47]. However, it may provide an even more potentiating effect on Treg which are highly dependent on IL-2 signaling for their survival and function.

When considering Treg homeostasis, such strategy could be especially relevant in context of ageing, a context of T cell lymphopenia in which IL-2 levels available are reduced. During ageing, proportions of Treg cells are increased [26]–[27]. It is tempting to speculate that IL-7 signaling could optimize the consumption of residual IL-2 and thus preserve their homeostasis. Importantly, increased proportion of CD25- Treg has been described during ageing, presumably due to the low availability in IL-2. Raynor et al., have suggested that Treg become dependent on other cytokines, namely IL-15 to compensate for the IL-2 deprived environment [27]. Whether IL-7 signaling could concomitantly limit the down-regulation of CD25 at aged Treg surface is currently under investigation. In addition to lymphopenic contexts, complementary IL-7 signaling could be involved in Treg maintenance in non-lymphoid tissues were IL-2 producing cells are present in limited numbers. In accordance, Gratz et al. have recently reported that skin infiltrating Treg are mainly dependent on IL-7 for their maintenance [39], as suggested by high CD127 expression at their surface [34], [39].

Demonstration of IL-7 dependent modulation of IL-2 signaling also provides a rationale for the association between T cell lymphopenia and Treg enrichment [48]–[49]. Despite not being absolutely necessary for Treg expansion in lymphopenic setting [9], IL-2 and lymphopenia have been shown to synergize in the induction of Treg expansion after transfer into empty hosts [10]. We studied the specific impact of IL-7 on purified Treg by transfer into empty hosts competent or not for IL-7 when co-injected with IL-2 thus providing the critical factor for Treg survival and expansion. Even in this context highly favorable for Treg expansion, we demonstrated *in vivo* that IL-7 availability influences the expansion of the Treg pool during IL-2 induced Treg proliferation. This observation provides a scientific background to protocols currently under clinical evaluation employing very low doses of IL-2 to induce selective expansion of Treg cells during immune-recovery from lymphopenia after hematopoietic stem cell transplantation [50]–[51].

IL-7 plays a critical role in conventional CD4+FOXP3- T cells proliferation and expansion upon transfer into lymphopenic hosts [52] and, similarly to what we reported for the Treg compartment, IL-7 modulates CD25 expression on activated conventional T cells [44]–[47]. We can therefore speculate that IL-7 effect on conventional T cells expansion in lymphopenic contexts could, at least partially, be mediated by its ability to sustain CD25 expression thus allowing to better perceive and transduce IL-2 signaling. This hypothesis merits investigation in future studies aiming to better understand the effects of IL-7 on conventional T cells homeostasis.

In addition to the impact of IL-7 on Treg homeostasis, IL-7 modulation CD25 expression at Treg surface could also affect Treg cells suppressive capacity. High CD25 expression allows Treg cells to efficiently bind and compete for IL-2 produced by conventional T cells during the early phases of immune responses [53] thus suppressing activated conventional T cells proliferation [12], [54] and survival [13]. By increasing CD25 expression at Treg surface, IL-7 could theoretically increase the competitive potential of Treg for IL-2 and therefore their suppressive activity. Intrinsic higher suppressive capacity of CD25+ compared to CD25- Treg fraction has not been previously established and remain uneasy to address: standard *in vitro* suppressive assay does not allow to conclude on intrinsic suppressive capacity because integrating survival and proliferation in addition to the strict intrinsic suppression capacity of each subsets. Similarly, *in vitro* suppressive assay in the presence or absence of IL-7 does not allow to directly ascertain the impact of IL-7 suppression because interfering both with conventional and Treg cells.

In summary, we established in the present report a role for IL-7 signaling in regulation of CD25 surface expression on Treg cells thus demonstrating an interplay between IL-2 and IL-7. In addition to provide IL-2 independent survival signals to Treg (as previously reported), IL-7 also constitutes an important cofactor for IL-2 induced expansion of Treg. IL-7 optimizes IL-2 consumption by Treg which may reveal extremely powerful to preserve Treg homeostasis.

## Supporting Information

S1 Figure **IL-7 signaling does not modulate CD122 expression on Treg cells** ***in vitro*** **.** (A) Total CD4+ cells isolated from FOXP3GFP mice were incubated overnight untreated (NT) or treated overnight with IL-2 (2 ng/ml) or IL-7 (10 ng/ml). (B) Representative FACS profiles of CD122 expression. CD122 expression on fresh (open histogram) and incubated (filled histogram) Foxp3/GFP+ cells and isotype control staining are shown. (C) MFI of CD122 expression on CD4+TCRβ+FOXP3+ cells after culture are shown. Mean ± SEM from three independent experiments are shown.
(TIF)

S2 Figure **STAT5 phosphorylation in FOXP3+ Treg and FOXP3- conventional CD4 T cells upon IL-2 exposure.** (A) Representative dot plot gated on TCRβ+ lymphocytes showing gating for CD4+ FOXP3- and FOXP3+ cells. (B) FACS profiles of phospho-STAT5 in FOXP3- (left panel) and FOXP3+ (right panel) cells either left untreated (unfilled histograms) or exposed to high doses IL-2 (10 ng/ml) (grey filled histograms). Isotype controls are indicated.
(TIF)
